# The New Superintendent

**Published:** 1892-01

**Authors:** 


### DR. FRANK S. WHITE, THE NEW SUPERINTENDENT.

On the 12th inst., the Board of Managers of the State Lunatic Asylum (Austin), appointed Dr. Frank S. White to be superintendent, to fill the vacancy caused by the death of Dr. Reeves.

This is a most excellent appointment. Dr. White has had as long, if not longer, experience in the actual care and treatment of the insane, and the management of asylum affairs than any man in Texas, unless we except Dr. D. R. Wallace. When the Terrell branch of the State asylum was completed (1885), Dr. White was selected for first assistant physician. He served two terms (4 years) in that capacity with Dr. Wallace, and when the present incumbent, Dr. Jno. Preston, was appointed superintendent *vice* Wallace, Dr. White was retained as first assistant physician, and was occupying that position at the time of his appointment as superintendent at Austin. He is a man of splendid physical and mental endowment, a learned physician, a close student, and moreover has demonstrated administrative and executive ability of high order.

Dr. White is in the prime of early but mature manhood; is about 32 years of age, and will make an excellent superintendent —a worthy successor to the lamented Reeves. For some years Dr. White's studies have been in this line, and he has written several articles on insanity. THE JOURNAL extends to him earnest and heartfelt congratulations on this deserved recognition of his claims to advancement, and this marked expression of confidence in his ability, and predicts for him a brilliant future.

BIOGRAPHY.—Frank Sprague White was born in Wise county, Texas, May 22, 1859; graduated in Indianapolis, Ind., at college physicians and surgeons, in 1884; in 1885 was appointed first assistant physician to asylum at Terrell, which position he has filled to date. In 1888, he was married to Miss Willie Daniel, of Austin, a niece of Gov. Jno. Ireland.

